# Association between self-reported body mass index and active life expectancy in a large community-dwelling sample of older U.S. adults

**DOI:** 10.1186/s12877-022-03021-7

**Published:** 2022-04-09

**Authors:** Haomiao Jia, Erica I. Lubetkin

**Affiliations:** 1 Department of Biostatistics, Mailman School of Public Health and School of Nursing, Columbia University, New York, NY USA; 2 Department of Community Health and Social Medicine, City University of New York School of Medicine, 160 Convent Avenue, HH313J, New York, NY 10031 USA

**Keywords:** Obesity, Ageing, Morbidity and mortality, Community-dwelling general population

## Abstract

**Background:**

Obesity may have a protective effect (greater survival) in older adults, a finding known as the “obesity paradox.” This study examined the association between self-reported body mass index (BMI) and active life expectancy (ALE) among older U.S. adults.

**Methods:**

Using the Medicare Health Outcomes Survey Cohort 15 (2012 baseline, 2014 follow-up), we estimated life expectancy and ALE by participants’ baseline BMI and age using multi-state models. A participant was classified as in an active state if this person reported having no difficulty for any of these six activities of daily living (ADLs).

**Results:**

Small differences in life expectancy were noted among persons in normal weight (BMI 18.5–24.9 kg/m^2^), overweight (BMI 25–29.9 kg/m^2^), and obesity ranges (BMI 30 kg/m^2^ and higher). However, persons with obesity had a significantly lower ALE. ALE at age 65 was 11.1 (11.0–11.2) years for persons with obesity, 1.2 (1.1–1.3) years less than that for the normal weight and overweight persons (12.3 years for both, 12.2–12.4). Persons with class III obesity had a significantly lower life expectancy and ALE than normal weight persons. Although persons with class I or II obesity had a similar life expectancy as normal weight persons, they have a shorter ALE.

**Conclusions:**

Although older adults with obesity have a similar life expectancy as normal weight persons, they have a significantly shorter ALE. Given the complex relationship of BMI and ALE, a “one size fits all” approach to weight management is not advisable.

**Supplementary Information:**

The online version contains supplementary material available at 10.1186/s12877-022-03021-7.

## Introduction

As the age structure in the United States changes, and the elderly population increases, the number of older adults with obesity will grow too [1]. In a recent recommendation statement, the U.S. Preventive Services Task Force (USPSTF) noted that obesity, defined as a body mass index (BMI) of 30 kg/m^2^ or more, is associated with both an increased risk for death and health problems, including having difficulties in activities of daily living (ADL) and disabilities, especially among adults younger than 65 years of age [2]. In noting research gaps, the USPSTF acknowledged that future research is needed to examine the effects of behavioral interventions for obesity on older adults aged 65 or older as well as persons who are overweight (BMI 25.0–29.9 kg/m^2^) given that, to date, few studies to have been conducted in this age group [2–4].

In the general population, the relationship between BMI and mortality resembles a J- or U-shaped curve [5]. Yet, in older persons, this relationship is more complex, as obesity may have a protective effect (greater survival), a finding known as the “obesity paradox” [6, 7]. Consensus exists that persons who are markedly underweight or severely obese have greater mortality than persons who are in between these two extremes. By contrast, the evidence is conflicting with regard to what range of BMI is associated with the greatest life expectancy, especially among older adults [4, 6–8]. Numerous investigations highlighted an attenuated relationship, or even a lack of a relationship, between obesity and mortality in older adults [4, 8]. For example, Reynolds and colleagues [4] found no difference in life expectancy at ages 70 years or older between persons with obesity and without obesity for both men and women.

By contrast, compared to normal weight persons, older adults with a BMI in the obese range are significantly more likely to have difficulties in ADLs or to become disabled [9]. Other investigators have noted greater obesity-associated morbidity in the context of a reduced active life expectancy (ALE) [4, 10–15] ALE, also called disability-free life expectancy (DFLE), is the number of future life years with no difficulty in performing ADLs (i.e., in “active” or non-disabled state). ALE is a good indicator of the overall long-term health of individuals, where greater ALE indicates a longer life span in full health [16, 17]. ALE differs from health-adjusted life expectancy (HALE) which is the number of years that a person can expect to live in full health [18]. Obesity, especially severe obesity, has been associated with a decreased ALE. For example, in contrast to life expectancy, Reynolds and colleagues [4] found a 1.4-year and 2.4-year decrease in ALE due to obesity for 70-year old men and women, respectively.

However, most of these studies estimated ALE by modeling transitions between active, inactive/disabled, and death states with BMI as one of the predictors [4, 10–14]. These analyses treated BMI as a permanent state and, therefore, the resultant life expectancy and ALE estimates were for persons whose obesity status would remain unchanged until death. To solve this problem, Diehr and colleagues [19] estimated transitions between different BMI-by-active states and estimated ALE for persons aged 65 and older by their baseline BMI-by-active states. However, this study found no association between baseline obesity status and decreased ALE among older adults, a result that differed from other studies.

The U.S. Medicare Health Outcome Survey (HOS) is the largest longitudinal survey of the U.S. community-dwelling Medicare beneficiaries. The primary aim of this study was to examine whether life expectancy and ALE differed by self-reported BMI among older U.S. adults from the Medicare HOS data. We estimated life expectancy and ALE every 2 years starting at age 65 for persons according to their baseline BMI. The multi-state model was used to allow participants’ BMI and active states to change during the remaining lifetime. The secondary aim was to identify the optimal BMI with the highest life expectancy and the highest ALE by examining life expectancy and ALE at different BMIs.

## Methods

The Medicare HOS is a nationwide annual survey of Medicare beneficiaries [20]. Each year, the HOS randomly selects a cohort of Medicare beneficiaries who enrolled in Medicare Advantage private health plans. The selected individuals are mailed a self-administered survey at baseline, and individuals who completed a baseline survey are resurveyed 2 years later. We used the Cohort 15 whose baseline data were collected in 2012 and follow-up data were collected in 2014. The HOS data were linked to the National Death Index. If a participant died by January 31, 2015, his/her date of death was included in the dataset. We included all respondents who were aged 65 years or older and alive at the baseline and participated in the baseline survey. The total sample was 164,597.

The HOS asks respondents to report their weight and height and calculates respondents’ body mass index (BMI) at the baseline and the follow-up [21]. We categorized BMI into underweight (< 18.5 kg/m^2^), normal weight (18.5–24.9 kg/m^2^), overweight (25–29.9 kg/m^2^), and obese (≥30.0 kg/m^2^). We also examined the following obesity subcategories: class I (30–34.9 kg/m^2^), class II (35–39.5 kg/m^2^), and class III obesity (≥ 40.0 kg/m^2^).

The HOS asks respondents whether they have difficulty with the following six basic ADLs: (1) bathing, (2) dressing, (3) eating, (4) getting in or out of chairs, (5) walking, and (6) using the toilet. These questions have been used for calculating ALE [4, 13, 16]. Each of these items (ADLs) has three possible responses: (1) “No, I do not have difficulty,” (2) “Yes, I have difficulty,” and (3) “I am unable to do this activity.” We used a definition of having a disability as answering (2) “Yes, I have difficulty” or (3) “I am unable to do this activity” for at least one of six ADLs. By contrast, a participant was classified as in an active state if this person reported (1) “No, I do not have difficulty” for all of these six ADLs [22]. Of note, these items also may be combined where persons are classified into five stages based on the severity of activity limitations [23, 24].

### Statistical analysis

Multi-state models were used to estimate life expectancy and ALE for cohorts of participants according to their baseline age and BMI [22, 23, 25]. Because the HOS data were collected at baseline and at follow-up after 2 years, we estimated life expectancy and ALE at ages 65, 67, …, etc. Suppose a multi-state model has *k* transient states *s* = (*s*_1_, *s*_2_, ⋯, *s*_*k*_) for *k* levels of BMI-by-active states (for example, “active and normal weight,” “dependent and normal weight,” “active and overweight,” “dependent and overweight,” etc.) and one absorbing state *s*_*k* + 1_ for dead. Let ${p}_t^{i,j}$ be probability in state *s*_*j*_ at age *t* + 2 among those in state *s*_*i*_ at age *t*.

The transition probabilities (${p}_t^{i,j},i,j\le k$) between different transient states were estimated from log-linear models with age as a time-dependent predictor, assuming a constant instantaneous transition rate in an age interval [22, 26]. The probability of death for each transient state (${p}_t^{i,k+1},i\le k$) during each age interval was estimated based on the probability of death for the total population and hazard ratio of death for each state relative to the reference group (active and normal weight) at different ages [22]. We used the probability of death from the 2012 U.S. life tables [27] as the probability of death for the total population and estimated hazard ratios using a Cox proportional hazard model with time-varying covariates from the HOS data [22, 26].

Life expectancy and ALE by participants’ baseline BMI was estimated by projecting number of remaining life years in each BMI-by-active states for an age cohort of persons in a given baseline BMI category. For a cohort of individuals with given numbers of persons in each states *i* at the starting age *x*, ${l}_x^i$, the expected numbers of persons in each state at ages *x* + 2, *x* + 4, …, can be obtained iteratively as ${l}_{t+2}^i={l}_t^i\left(1-\sum_{j=1,j\ne i}^{k+1}{p}_t^{i,j}\right)+\sum_{j=1,j\ne i}^k{l}_t^j{p}_t^{j,i}$. Let ${L}_t^i$ be number of years lived in state *s*_*i*_ during the age interval from *t* to *t* + 2 for the age cohort. The expected number of remaining life years in state *s*_*i*_ for this age cohort is ${e}_x^i=\left({\Sigma}_{t\ge x}\kern0.5em {L}_t^i\right)/{l}_x$ where ${l}_x=\sum_{i=1}^k{l}_x^i$ is the total number of persons at the starting age *x*. Therefore, life expectancy for this age cohort is ${\sum}_{i=1}^k{e}_x^i.$ Let *s*_*A*_ be the set for all active states (“active and normal weight”, “active and overweight,” etc.), ALE for this age cohort is the summation of ${e}_x^i$ over all active states, $\mathrm{ALE}=\sum_{i\in {s}_A}{e}_x^i$.

Observations with missing data for BMI and ADL were excluded in multivariable analysis (about 6% at baseline and 11% at follow-up). Standard errors of life expectancy and ALE estimates were estimated using the bootstrap method with 1000 replications [22, 26].

## Results

At baseline, the average participant age was 75.1 years (Table 1). Women comprised 58% of the sample, and white non-Hispanics comprised 76% of the sample. About 31% had a BMI in the normal range, 38% in the overweight range, 29% in the obesity range, and only 2% in the underweight range. Within the subcategories of obesity, 18% were class I obesity, 7% were class II obesity, and 4% were class III obesity. Similar BMI distributions were reported at the follow-up survey.

**Table 1:** Sample Characteristics at Baseline and Follow-up

	Baseline 2012		Follow-up 2014	
	N	percent	N	percent
Total sample	164,597	100%	100,290	100%
Age, Mean (SD)	75.1 (7.4)		76.2 (6.7)	
65–74	87,972	53.4%	47,929	47.8%
75–84	55,676	33.8%	39,337	39.2%
85–94	19,313	11.7%	12,308	12.3%
95+	1636	1.0%	716	0.7%
Female	95,115	57.8%	58,519	58.4%
Race/ethnicity				
White non-Hispanics	121,334	76.1%	77,694	78.2%
Black non-Hispanics	13,031	8.2%	7427	7.5%
Hispanics	15,735	9.9%	8803	8.9%
Other	9404	5.9%	5408	5.4%
BMI categories				
Underweight (< 18.5 kg/m^2^)	3274	2.1%	1602	1.8%
Normal (18.5–24.9 kg/m^2^)	48,402	31.1%	28,246	30.9%
Overweight (25–29.9 kg/m^2^)	59,201	38.0%	35,261	38.6%
Obesity (≥30 kg/m^2^)	44,877	28.8%	26,238	28.7%
Class I obesity (30–34.9 kg/m^2^)	28,783	18.5%	17,033	18.6%
Class II obesity (35–39.9 kg/m^2^)	10,283	6.6%	5978	6.5%
Class III obesity (≥40 kg/m^2^)	5811	3.7%	3227	3.5%
Any Difficulty with ADL				
No	100,475	62.4%	62,680	66.7%
Yes	60,523	37.6%	31,298	33.3%

Table 2 presents life expectancy and ALE by baseline BMI categories. For example, the average number of years of life remaining is 19.2 years for a 65-year old person with obesity. Of these 19.2 years, 11.1 years were in “active” state and 8.1 (=19.2–11.1) years were in “dependent” state. Participants whose BMI was in the underweight category had a lower life expectancy and ALE than those whose BMIs were in the normal weight, overweight, and obese categories, both for the total sample and for men and women. Because only 2% of respondents were underweight, the remainder of this manuscript will focus on those whose BMI ≥18.5 kg/m^2^.

**Table 2:** Life Expectancy and Active Life Expectancy by Initial BMI at Different Ages, U.S. Older Adults

Age	Life expectancy							Active life expectancy						
	under weight	normal weight	over weight	obesity	obesity subclass			under weight	normal weight	over weight	obesity	obesity subclass		
					I	II	III					I	II	III
65	16.9	19.1	19.6	19.2	19.5	19.1	18.3	10.3	12.3	12.3	11.1	11.8	11.3	9.7
67	15.2	17.5	18.1	17.7	18.0	17.6	16.7	9.1	11.1	11.2	10.0	10.7	10.1	8.7
69	13.7	16.1	16.6	16.2	16.5	16.1	15.2	8.0	9.9	10.0	8.9	9.5	9.0	7.6
71	12.2	14.6	15.2	14.8	15.0	14.7	13.7	6.9	8.8	8.9	7.8	8.4	7.9	6.7
73	10.9	13.3	13.8	13.4	13.7	13.4	12.4	5.9	7.7	7.8	6.8	7.3	6.8	5.7
75	9.7	11.9	12.5	12.1	12.3	12.0	11.2	5.0	6.7	6.7	5.8	6.3	5.8	4.9
77	8.6	10.7	11.2	10.9	11.0	10.8	10.0	4.2	5.7	5.7	4.9	5.3	4.9	4.1
79	7.6	9.5	10.0	9.7	9.8	9.6	8.9	3.5	4.8	4.8	4.0	4.3	4.0	3.4
81	6.6	8.4	8.9	8.6	8.7	8.5	7.9	2.8	4.0	3.9	3.2	3.5	3.2	2.7
83	5.8	7.4	7.8	7.5	7.6	7.5	7.0	2.3	3.2	3.2	2.6	2.8	2.6	2.2
85	5.0	6.5	6.9	6.6	6.6	6.5	6.1	1.8	2.6	2.5	2.0	2.1	2.0	1.7
87	4.4	5.7	6.0	5.7	5.7	5.7	5.3	1.3	2.0	1.9	1.5	1.6	1.5	1.3
89	3.8	4.9	5.2	4.9	4.9	4.9	4.5	1.0	1.6	1.5	1.1	1.2	1.1	1.0
91	3.3	4.3	4.5	4.2	4.2	4.2	3.8	0.7	1.2	1.1	0.8	0.9	0.8	0.8
93	2.8	3.8	3.9	3.6	3.6	3.6	3.2	0.5	0.9	0.8	0.6	0.6	0.6	0.6
95	2.4	3.3	3.4	3.0	3.1	3.1	2.6	0.3	0.6	0.6	0.4	0.4	0.5	0.4

Standard errors of estimates are available in eTable 1

Overall, the differences in life expectancies were relatively small (≤ 0.5 years), but still statistically significant (standard errors in supplementary file: eTable 1), among persons whose BMI was in the normal weight, overweight, and obese range. However, persons with obesity had a much lower ALE than normal weight and overweight persons. For example, ALE at age 65 was 11.1 years for the persons with obesity, 1.2 years less than that for the normal weight and overweight persons (both were 11.3 years). The same patterns were consistent throughout the range of ages, and the differences among these three groups were even smaller in older ages.

Although similar results were observed for men and women (Table 3), some small differences emerged. For example, men with obesity had a slightly higher life expectancy than normal weight men, while men with obesity had a slightly lower ALE than normal weight men. By contrast, women with obesity had a similar life expectancy as normal weight women, while women with obesity had a much lower ALE than normal weight women.

**Table 3:** Life Expectancy and Active Life Expectancy by Initial BMI at Different Ages, U.S. Older Men and Women

Age	Life expectancy							Active life expectancy						
	under weight	normal weight	over weight	Obesity	obesity subclass			under weight	normal weight	over weight	obesity	obesity subclass		
					I	II	III					I	II	III
Men														
65	16.0	17.3	18.3	17.9	18.2	17.7	16.5	10.1	11.4	12.1	11.0	11.6	10.9	9.2
67	14.2	15.9	16.9	16.4	16.7	16.3	15.0	8.8	10.4	11.0	9.9	10.5	9.8	8.3
69	12.5	14.5	15.4	15.0	15.3	14.9	13.6	7.6	9.3	9.9	8.9	9.4	8.7	7.4
71	11.0	13.2	14.1	13.7	13.9	13.5	12.4	6.5	8.2	8.8	7.8	8.3	7.7	6.5
73	9.6	11.9	12.8	12.4	12.5	12.2	11.2	5.5	7.2	7.7	6.8	7.2	6.6	5.7
75	8.5	10.7	11.5	11.1	11.3	11.0	10.2	4.6	6.3	6.6	5.8	6.1	5.7	4.9
77	7.4	9.5	10.3	9.9	10.1	9.8	9.1	3.8	5.3	5.7	4.9	5.1	4.8	4.1
79	6.5	8.4	9.2	8.8	8.9	8.7	8.2	3.1	4.5	4.7	4.0	4.2	3.9	3.4
81	5.7	7.4	8.1	7.7	7.8	7.6	7.3	2.5	3.8	3.9	3.3	3.4	3.2	2.8
83	4.9	6.5	7.1	6.7	6.8	6.6	6.3	2.0	3.1	3.2	2.6	2.7	2.5	2.2
85	4.2	5.7	6.2	5.8	5.9	5.7	5.4	1.6	2.5	2.5	2.0	2.1	2.0	1.7
87	3.6	5.0	5.4	5.0	5.0	4.9	4.6	1.2	2.0	2.0	1.6	1.6	1.5	1.3
89	3.1	4.3	4.7	4.2	4.3	4.2	3.7	0.9	1.6	1.5	1.2	1.3	1.1	0.9
91	2.7	3.8	4.0	3.6	3.6	3.5	2.9	0.7	1.3	1.2	0.9	1.0	0.8	0.7
93	2.3	3.3	3.5	3.0	3.0	3.0	2.2	0.5	1.0	0.9	0.7	0.7	0.6	0.5
95	1.9	2.9	3.0	2.5	2.5	2.5	1.6	0.4	0.8	0.7	0.5	0.6	0.5	0.3
Women														
65	17.7	20.4	20.7	20.3	20.6	20.3	19.5	10.6	12.9	12.5	11.2	12.1	11.5	10.0
67	16.1	18.8	19.1	18.7	19.0	18.7	17.8	9.4	11.6	11.3	10.1	10.9	10.4	8.9
69	14.5	17.3	17.6	17.2	17.5	17.1	16.1	8.2	10.4	10.2	9.0	9.7	9.2	7.8
71	13.1	15.7	16.1	15.7	16.0	15.6	14.6	7.1	9.2	9.0	7.9	8.5	8.0	6.8
73	11.7	14.3	14.7	14.2	14.5	14.2	13.1	6.1	8.1	7.9	6.8	7.4	6.9	5.8
75	10.4	12.9	13.3	12.8	13.1	12.8	11.8	5.2	7.0	6.8	5.8	6.3	5.9	4.9
77	9.2	11.5	11.9	11.5	11.7	11.4	10.5	4.4	6.0	5.8	4.9	5.3	4.9	4.1
79	8.1	10.2	10.7	10.2	10.4	10.2	9.4	3.6	5.0	4.8	4.0	4.4	4.0	3.4
81	7.1	9.0	9.5	9.0	9.2	9.0	8.3	2.9	4.1	4.0	3.2	3.6	3.3	2.7
83	6.2	7.9	8.3	7.9	8.1	7.9	7.3	2.3	3.3	3.2	2.6	2.8	2.6	2.2
85	5.3	6.9	7.3	6.9	7.0	6.9	6.3	1.8	2.6	2.5	2.0	2.1	2.0	1.7
87	4.6	6.0	6.3	6.0	6.0	5.9	5.5	1.4	2.0	1.9	1.5	1.6	1.5	1.3
89	4.0	5.2	5.5	5.1	5.2	5.1	4.7	1.0	1.5	1.5	1.1	1.2	1.1	1.0
91	3.4	4.5	4.7	4.4	4.4	4.4	4.0	0.7	1.1	1.1	0.8	0.8	0.8	0.7
93	3.0	3.9	4.1	3.7	3.8	3.8	3.3	0.5	0.8	0.8	0.6	0.6	0.6	0.5
95	2.6	3.4	3.5	3.1	3.2	3.2	2.7	0.3	0.6	0.6	0.4	0.4	0.4	0.4

Standard errors of estimates are available in eTable 1

For the three subcategories of obesity (class I, II, and III), both life expectancy and ALE decreased with level of obesity. Compared to those whose BMI was in the normal range, older adults with class III obesity had a significantly lower life expectancy and ALE. Yet, although persons with class I or II obesity had a similar life expectancy as normal weight persons, they have a shorter ALE.

**Fig. 1:**
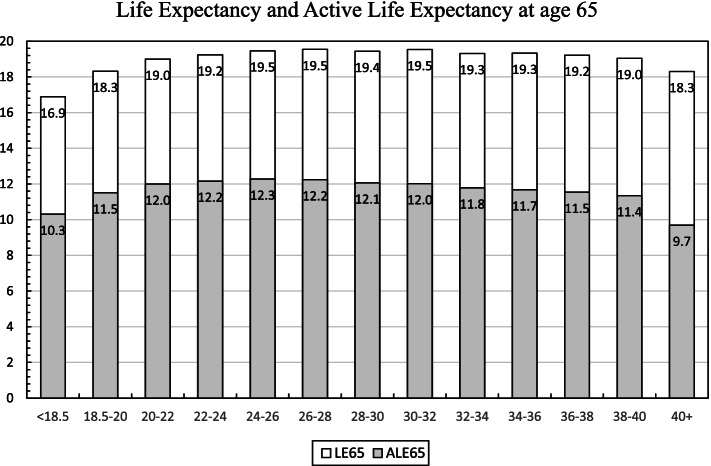
Life Expectancy and Active Life Expectancy at Age 65 Years by BMI. LE65: life expectancy at age 65 years. ALE65: active life expectancy at age 65 years. Standard errors of estimates are available in eTable 2

Figure 1 presents distributions of life expectancy and ALE at age 65 at different BMIs, with standard errors in supplementary file (eTable 2). Overall, life expectancy increased constantly with BMI from < 18.5 to 24 kg/m^2^, then stayed relatively stable at approximately 19.5 years for a BMI from 24 to 32 kg/m^2^, and started to decline when BMI ≥ 32 kg/m^2^. Similarly, ALE increased with a BMI from < 18.5 to 22 kg/m^2^, then stayed stable for a BMI from 22 to 28 kg/m^2^, and started to decline when BMI ≥ 28 kg/m^2^. Similar patterns were observed for men and women (eFigure 1).

## Discussion

Consistent with past investigations [4, 8, 10–14, 19, 28], our results indicate that older adults with obesity have a similar life expectancy as do normal weight older persons, a finding that provides further evidence for the “obesity paradox.” By contrast, our results show the adverse impact of obesity on morbidity, as older adults with obesity have a shorter ALE as compared to normal weight persons. With regard to subcategories of obesity, participants with class III obesity had a significantly lower life expectancy than those whose BMI was in the normal or overweight range [2], while the impact of class I or class II obesity on life expectancy was either very small or nonexistent. However, in addition to persons with class III obesity, persons with class I or class II obesity had a significantly lower ALE compared to normal weight persons [3, 4, 29].

Our results reinforce the complex relationship between BMI and both life expectancy and ALE in the U.S. older population that has been noted by previous investigators [4, 10–14, 19]. A given BMI may be associated with the greatest life expectancy while a different BMI may be associated with the greatest ALE. The optimal BMI for having the highest life expectancy ranged from 24 to 32 kg/m^2^, and the optimal BMI for having the highest ALE ranged from 22 to 28 kg/m^2^.

Some differences were observed among subgroups categorized as having a normal BMI. Life expectancy among older adults with a BMI between 18.5 and 20 kg/m^2^ was similar to those with class III obesity. However, ALE for this group was greater than that for those with class III obesity. These results are consistent with the findings by Diehr and colleagues [30]; these investigators found that a BMI between 18.5 and 20 kg/m^2^ among older adults was associated with higher mortality and fewer years of healthy life as compared to a BMI from 20 to 25 kg/m^2^. Our study also found that life expectancy among older adults with a BMI between 20 and 24 kg/m^2^ was similar to that of older adults with class II obesity, but ALE was higher than the ALE of older adults with class I and II obesity. Such results provide further evidence that a BMI at the low normal range might be “too low” for older adults, especially in light of the weaker evidence connecting obesity with increased mortality. Other investigators have noted an increase in all-cause mortality risk for older adults with a BMI at the lower end of the normal range, beginning with a BMI of less than 23.0 kg/m^2^ [19, 31–33].

Many studies have found that obesity, especially severe obesity, is associated with a significantly decreased ALE among older adults [4, 10–14]. However, most of these studies treated BMI as a permanent state. In other words, ALE estimates were for participants whose obesity status would remain unchanged until death (i.e., comparing ALE between those who were obese for their entire remaining lifetime vs. those who were a normal weight for their entire remaining lifetime). To our knowledge, only the study by Diehr and colleagues [19] estimated ALE for older persons according to their baseline BMI by estimating transitions between different BMI categories during the remainder of their lifetime. However, this study found no association between baseline obesity status and decreased ALE among older adults. Yet, Diehr’s study had a number of weaknesses, including that the sample size (5888) was too small to provide reliable estimates and was comprised of seniors from four U.S. counties in California, as opposed to a representative sample of the U.S. general population [19].

Our analyses add to the literature by determining the difference in life expectancy and ALE according to participants’ baseline BMI in a large longitudinal sample of the U.S. community-dwelling elderly population. The multi-state modeling method enabled us to examine multiple and recurrent events simultaneously. The method also allowed for a transferring between different states for both BMI and active states during the remaining life years [19]. The large sample size of the HOS permitted us to estimate life expectancy and ALE in small BMI subcategories with good reliability. The standard errors of estimates were mostly less than 0.1 years (see eTables 1 and 2).

Our study has a number of limitations. First, because this analysis used data from the Medicare HOS, a survey of Medicare beneficiaries who voluntarily enrolled in private Medicare Advantage health plans, this sample may be younger and healthier than the overall Medicare population [34]. Second, potential bias might exist due to non-participation in the follow-up survey as, for example, respondents now might be institutionalized. While no differences in baseline characteristics, including age, sex, ADL, BMI, and chronic conditions, were noted between persons who participated and persons who did not participate in the follow-up survey, whites were more likely to complete the follow-up survey as compared to other racial/ethnic groups (see Table 3). Third, BMI was calculated based on self-reported weight and height. Obese persons may be more likely to under-report their weights and over-report their heights compared to non-obese persons, and men and elderly women also may be more likely to over-report their heights [35–37]. Because these reporting patterns would result in misclassification due to estimates of obesity’s impact biased toward the null, this might partially explain the lack of a difference in estimates between older persons with obesity and normal weight persons. A recent study has highlighted the importance of including measurements of height and weight in large data sets, given the presence of non-classical error in self-reported BMI [38]. Fourth, respondents reported their own limitations in their ADLs, which were not validated by medical chart reviews. Certain ADL items may have a wider interpretation due to such factors as culture, education, and language. Fifth, BMI might not be the most accurate way to measure obesity and waist circumference might provide additional information regarding obesity-related health risk in the elderly, given that waist circumference has been shown to impact mortality at all levels of BMI [39].

## Conclusions

In 2011, the U.S. Centers for Medicare & Medicaid Services introduced a Medicare obesity counseling benefit based on the USPSTF’s recommendation of screening for obesity in adults [40]. Persons with a BMI ≥ 30 kg/m^2^ would be eligible for Intensive Behavioral Therapy in primary care. However, as noted, evidence of the benefits of counseling for weight loss was based on studies of younger persons. Given the concern of losing muscle and increasing in the elderly, recommended strategies to promote weight loss might differ by age [41]. Fewer trials have evaluated the effectiveness of weight loss interventions in older adults where approximately one-quarter of all weight lost is fat-free mass [42]. Clinicians should be more cautious about relying solely on BMI to make recommendations about weight loss. In the elderly, loss of fat-free mass contributes to sarcopenia, and sarcopenia is associated with disability and frailty [43, 44].

Given the complex relationship of BMI with life expectancy and active life expectancy, a “one size fits all” approach to weight management is not advisable. Instead, a deeper understanding of the relationship between obesity and other co-morbidities, in addition to health-related quality of life, and physical function should be sought. The Medicare Health Outcomes Survey data provide an opportunity to monitor the health of the U.S. community-dwelling older population, a growing population that traditionally has been overlooked in the literature. Quantifying life expectancy and active life expectancy according to a person’s BMI enables a more holistic portrait of the effect of weight on individual and population health.

## Supplementary Information

**Additional file 1: eTable 1.** Standard Error (S.E.) of Estimates in Tables 2 and 3. **eTable 2.** Standard Error (S.E.) of Estimates in Fig. 1 and **eFigure 1.** **eTable 3.** Characteristics of Persons who Completed and Did Not Complete Follow-Up Survey. **eFigure 1.** Life Expectancy and Active Life Expectancy at Age 65 Years by BMI, Men and Women. LE65: life expectancy at age 65 years. ALE65: active life expectancy at age 65 years. Standard errors of estimates are available in supplemental data file.

## Data Availability

This study is a secondary data analysis using the Limited Data Set (LDS) of the HOS from the U.S. Centers for Medicare & Medicaid Services (CMS). The dataset contains potentially identifying or sensitive patient information (e.g., participants’ zip code, date of birth, date of death, etc.). A signed Data Use Agreement (DUA) with CMS is required to obtain LDS data files (https://www.cms.gov/Research-Statistics-Data-and-Systems/Files-for-Order/LimitedDataSets/HOS ). In order to request a LDS files, investigators must follow the instructions on this link: https://www.cms.gov/Research-Statistics-Data-and-Systems/Files-for-Order/Data-Disclosures-Data-Agreements/EPPEpilot-LDSS
