# Supplementary material for: Association between self-reported body mass index and active life expectancy in a large community-dwelling sample of older U.S. adults
Source: BMC Geriatr. 2022 Apr 9;22:310. doi: 10.1186/s12877-022-03021-7 (PMC8994875; doi:10.1186/s12877-022-03021-7)
Supplement: Supplementary file 1 — Additional file 1: eTable 1. Standard Error (S.E.) of Estimates in Tables 2 and 3. eTable 2. Standard Error (S.E.) of Estimates in Fig. 1 and eFigure 1. eTable 3. Characteristics of Persons who Completed and Did Not Complete Follow-Up Survey. eFigure 1. Life Expectancy and Active Life Expectancy at Age 65 Years by BMI, Men and Women. LE65: life expectancy at age 65 years. ALE65: active life expectancy at age 65 years. Standard errors of estimates are available in supplemental data file. [file 12877_2022_3021_MOESM1_ESM.docx]

Supplementary file: 3 tables, 1 figure

eTable 1. Standard Error (S.E.) of Estimates in Tables 2 and 3

eTable 2. Standard Error (S.E.) of Estimates in Figures 1 and eFigure 1

eTable 3: Characteristics of Persons who Completed and Did Not Complete Follow-Up Survey

eFigure 1. Life Expectancy and Active Life Expectancy at Age 65 Years by BMI, Men and Women

LE65: life expectancy at age 65 years

ALE65: active life expectancy at age 65 years

Standard errors of estimates are available in supplemental data file.

eTable 1. Standard Error (S.E.) of Estimates in Tables 2 and 3

|  | Life expectancy | | | | | | | Active life expectancy | | | | | | |
| --- | --- | --- | --- | --- | --- | --- | --- | --- | --- | --- | --- | --- | --- | --- |
| Age | under  weight | normal  weight | over  weight | obesity | obesity subgroups | | | under  weight | normal  weight | over  weight | obesity | obesity subgroups | | |
|  |  |  |  |  | I | II | III |  |  |  |  | I | II | III |
| Total sample | | | | | | | | | | | | | | |
| 65 | 0.26 | 0.04 | 0.03 | 0.03 | 0.03 | 0.06 | 0.10 | 0.19 | 0.04 | 0.03 | 0.03 | 0.03 | 0.05 | 0.09 |
| 67 | 0.20 | 0.03 | 0.02 | 0.03 | 0.03 | 0.05 | 0.10 | 0.14 | 0.03 | 0.03 | 0.03 | 0.03 | 0.04 | 0.07 |
| 69 | 0.18 | 0.03 | 0.02 | 0.03 | 0.03 | 0.06 | 0.10 | 0.12 | 0.03 | 0.02 | 0.03 | 0.03 | 0.04 | 0.08 |
| 71 | 0.17 | 0.03 | 0.02 | 0.03 | 0.03 | 0.06 | 0.11 | 0.11 | 0.03 | 0.02 | 0.03 | 0.03 | 0.04 | 0.08 |
| 73 | 0.17 | 0.03 | 0.02 | 0.03 | 0.03 | 0.06 | 0.11 | 0.10 | 0.03 | 0.02 | 0.03 | 0.03 | 0.04 | 0.07 |
| 75 | 0.15 | 0.03 | 0.02 | 0.03 | 0.03 | 0.06 | 0.11 | 0.09 | 0.03 | 0.02 | 0.03 | 0.03 | 0.04 | 0.07 |
| 77 | 0.14 | 0.03 | 0.02 | 0.03 | 0.03 | 0.07 | 0.12 | 0.08 | 0.03 | 0.02 | 0.03 | 0.03 | 0.04 | 0.07 |
| 79 | 0.12 | 0.03 | 0.02 | 0.04 | 0.04 | 0.08 | 0.12 | 0.07 | 0.03 | 0.02 | 0.03 | 0.03 | 0.04 | 0.07 |
| 81 | 0.11 | 0.03 | 0.02 | 0.04 | 0.04 | 0.09 | 0.13 | 0.06 | 0.03 | 0.02 | 0.03 | 0.03 | 0.04 | 0.07 |
| 83 | 0.10 | 0.03 | 0.02 | 0.04 | 0.05 | 0.10 | 0.14 | 0.05 | 0.03 | 0.02 | 0.03 | 0.03 | 0.04 | 0.07 |
| 85 | 0.10 | 0.03 | 0.02 | 0.05 | 0.05 | 0.10 | 0.15 | 0.05 | 0.02 | 0.02 | 0.03 | 0.03 | 0.04 | 0.06 |
| 87 | 0.10 | 0.03 | 0.03 | 0.05 | 0.06 | 0.11 | 0.16 | 0.04 | 0.02 | 0.02 | 0.03 | 0.03 | 0.04 | 0.06 |
| 89 | 0.09 | 0.03 | 0.03 | 0.06 | 0.06 | 0.12 | 0.17 | 0.04 | 0.02 | 0.02 | 0.03 | 0.03 | 0.04 | 0.06 |
| 91 | 0.09 | 0.03 | 0.03 | 0.06 | 0.07 | 0.14 | 0.19 | 0.03 | 0.02 | 0.02 | 0.03 | 0.03 | 0.04 | 0.06 |
| 93 | 0.10 | 0.03 | 0.04 | 0.08 | 0.08 | 0.18 | 0.21 | 0.03 | 0.02 | 0.02 | 0.03 | 0.03 | 0.04 | 0.06 |
| 95 | 0.10 | 0.03 | 0.06 | 0.10 | 0.10 | 0.22 | 0.24 | 0.03 | 0.03 | 0.03 | 0.03 | 0.03 | 0.05 | 0.06 |
| Men | | | | | | | | | | | | | | |
| 65 | 0.40 | 0.07 | 0.04 | 0.05 | 0.05 | 0.11 | 0.21 | 0.31 | 0.06 | 0.04 | 0.05 | 0.05 | 0.09 | 0.17 |
| 67 | 0.33 | 0.06 | 0.03 | 0.04 | 0.04 | 0.09 | 0.19 | 0.24 | 0.05 | 0.04 | 0.04 | 0.04 | 0.07 | 0.14 |
| 69 | 0.29 | 0.06 | 0.03 | 0.04 | 0.05 | 0.10 | 0.20 | 0.20 | 0.05 | 0.03 | 0.04 | 0.04 | 0.07 | 0.14 |
| 71 | 0.28 | 0.06 | 0.03 | 0.05 | 0.05 | 0.11 | 0.20 | 0.19 | 0.05 | 0.03 | 0.04 | 0.04 | 0.08 | 0.13 |
| 73 | 0.27 | 0.05 | 0.03 | 0.05 | 0.05 | 0.11 | 0.20 | 0.18 | 0.05 | 0.03 | 0.04 | 0.04 | 0.07 | 0.13 |
| 75 | 0.26 | 0.05 | 0.03 | 0.05 | 0.05 | 0.11 | 0.21 | 0.16 | 0.04 | 0.03 | 0.04 | 0.04 | 0.07 | 0.13 |
| 77 | 0.24 | 0.05 | 0.03 | 0.05 | 0.05 | 0.12 | 0.22 | 0.14 | 0.04 | 0.03 | 0.04 | 0.04 | 0.08 | 0.14 |
| 79 | 0.22 | 0.05 | 0.03 | 0.06 | 0.06 | 0.14 | 0.25 | 0.12 | 0.04 | 0.03 | 0.04 | 0.04 | 0.08 | 0.14 |
| 81 | 0.20 | 0.05 | 0.03 | 0.07 | 0.07 | 0.16 | 0.27 | 0.11 | 0.04 | 0.03 | 0.04 | 0.05 | 0.08 | 0.15 |
| 83 | 0.19 | 0.05 | 0.03 | 0.07 | 0.08 | 0.18 | 0.30 | 0.10 | 0.04 | 0.03 | 0.05 | 0.05 | 0.09 | 0.15 |
| 85 | 0.19 | 0.05 | 0.04 | 0.08 | 0.09 | 0.20 | 0.36 | 0.09 | 0.04 | 0.03 | 0.05 | 0.05 | 0.09 | 0.16 |
| 87 | 0.19 | 0.05 | 0.04 | 0.09 | 0.10 | 0.22 | 0.47 | 0.08 | 0.04 | 0.03 | 0.05 | 0.05 | 0.10 | 0.18 |
| 89 | 0.19 | 0.05 | 0.04 | 0.11 | 0.11 | 0.27 | 0.60 | 0.08 | 0.04 | 0.04 | 0.06 | 0.06 | 0.10 | 0.20 |
| 91 | 0.20 | 0.05 | 0.05 | 0.13 | 0.14 | 0.34 | 0.74 | 0.08 | 0.04 | 0.04 | 0.06 | 0.07 | 0.11 | 0.21 |
| 93 | 0.21 | 0.05 | 0.06 | 0.17 | 0.18 | 0.42 | 0.83 | 0.08 | 0.05 | 0.04 | 0.07 | 0.08 | 0.12 | 0.21 |
| 95 | 0.22 | 0.06 | 0.08 | 0.21 | 0.23 | 0.51 | 0.83 | 0.08 | 0.05 | 0.05 | 0.08 | 0.10 | 0.13 | 0.20 |
| Women | | | | | | | | | | | | | | |
| 65 | 0.34 | 0.04 | 0.03 | 0.04 | 0.04 | 0.08 | 0.12 | 0.23 | 0.05 | 0.04 | 0.05 | 0.05 | 0.06 | 0.10 |
| 67 | 0.25 | 0.04 | 0.03 | 0.04 | 0.04 | 0.06 | 0.11 | 0.16 | 0.04 | 0.04 | 0.04 | 0.04 | 0.05 | 0.09 |
| 69 | 0.21 | 0.04 | 0.03 | 0.04 | 0.04 | 0.07 | 0.12 | 0.13 | 0.04 | 0.03 | 0.04 | 0.04 | 0.05 | 0.09 |
| 71 | 0.20 | 0.04 | 0.03 | 0.04 | 0.04 | 0.07 | 0.13 | 0.12 | 0.04 | 0.03 | 0.04 | 0.04 | 0.05 | 0.09 |
| 73 | 0.19 | 0.04 | 0.03 | 0.04 | 0.04 | 0.07 | 0.13 | 0.11 | 0.04 | 0.03 | 0.04 | 0.04 | 0.05 | 0.08 |
| 75 | 0.18 | 0.04 | 0.03 | 0.04 | 0.04 | 0.08 | 0.13 | 0.10 | 0.04 | 0.03 | 0.04 | 0.04 | 0.05 | 0.08 |
| 77 | 0.16 | 0.04 | 0.03 | 0.04 | 0.05 | 0.08 | 0.13 | 0.09 | 0.03 | 0.03 | 0.04 | 0.04 | 0.05 | 0.08 |
| 79 | 0.14 | 0.04 | 0.03 | 0.05 | 0.05 | 0.09 | 0.14 | 0.08 | 0.03 | 0.03 | 0.03 | 0.03 | 0.05 | 0.08 |
| 81 | 0.13 | 0.04 | 0.03 | 0.05 | 0.05 | 0.10 | 0.15 | 0.07 | 0.03 | 0.03 | 0.03 | 0.03 | 0.05 | 0.08 |
| 83 | 0.12 | 0.04 | 0.03 | 0.05 | 0.05 | 0.11 | 0.16 | 0.06 | 0.03 | 0.03 | 0.03 | 0.03 | 0.05 | 0.07 |
| 85 | 0.11 | 0.04 | 0.03 | 0.06 | 0.06 | 0.12 | 0.17 | 0.05 | 0.03 | 0.03 | 0.03 | 0.03 | 0.05 | 0.07 |
| 87 | 0.11 | 0.04 | 0.04 | 0.06 | 0.07 | 0.13 | 0.18 | 0.05 | 0.03 | 0.03 | 0.03 | 0.03 | 0.05 | 0.07 |
| 89 | 0.11 | 0.04 | 0.04 | 0.07 | 0.08 | 0.14 | 0.19 | 0.04 | 0.03 | 0.03 | 0.03 | 0.03 | 0.05 | 0.06 |
| 91 | 0.11 | 0.04 | 0.05 | 0.07 | 0.08 | 0.16 | 0.21 | 0.04 | 0.03 | 0.03 | 0.03 | 0.03 | 0.05 | 0.06 |
| 93 | 0.11 | 0.04 | 0.05 | 0.09 | 0.09 | 0.20 | 0.24 | 0.04 | 0.03 | 0.03 | 0.03 | 0.03 | 0.05 | 0.06 |
| 95 | 0.12 | 0.04 | 0.07 | 0.11 | 0.11 | 0.25 | 0.28 | 0.03 | 0.03 | 0.03 | 0.03 | 0.03 | 0.05 | 0.06 |

eFigure 1. Life Expectancy and Active Life Expectancy at Age 65 Years by BMI, Men and Women

LE65: life expectancy at age 65 years

ALE65: active life expectancy at age 65 years

Standard errors of estimates are available in supplemental data file

eTable 2. Standard Error (S.E.) of Estimates in Figures 1 and eFigure 1

|  | Total | | Men | | Women | |
| --- | --- | --- | --- | --- | --- | --- |
| BMI | LE65 | ALE65 | LE65 | ALE65 | LE65 | ALE65 |
| <18.5 | 0.25 | 0.17 | 0.37 | 0.29 | 0.33 | 0.22 |
| 18.5-20 | 0.15 | 0.11 | 0.35 | 0.25 | 0.15 | 0.11 |
| 20-22 | 0.08 | 0.06 | 0.18 | 0.14 | 0.08 | 0.07 |
| 22-24 | 0.05 | 0.05 | 0.09 | 0.08 | 0.06 | 0.06 |
| 24-26 | 0.04 | 0.04 | 0.06 | 0.06 | 0.05 | 0.05 |
| 26-28 | 0.04 | 0.03 | 0.05 | 0.05 | 0.05 | 0.05 |
| 28-30 | 0.04 | 0.04 | 0.06 | 0.05 | 0.05 | 0.05 |
| 30-32 | 0.04 | 0.04 | 0.06 | 0.06 | 0.05 | 0.05 |
| 32-34 | 0.06 | 0.05 | 0.09 | 0.07 | 0.07 | 0.06 |
| 34-36 | 0.07 | 0.06 | 0.11 | 0.09 | 0.08 | 0.07 |
| 36-38 | 0.09 | 0.07 | 0.15 | 0.12 | 0.10 | 0.08 |
| 38-40 | 0.11 | 0.08 | 0.17 | 0.13 | 0.14 | 0.10 |
| 40+ | 0.10 | 0.09 | 0.21 | 0.17 | 0.11 | 0.10 |

eTable 3: Characteristics of Persons who Completed and Did Not Complete Follow-Up Survey

|  | Completed Follow-Up Survey (N=100,290) | Did Not Complete Follow-Up Survey (N=38,196) |
| --- | --- | --- |
|  | Mean or Percent | Mean |
| Age | 74.1 | 74.2 |
| Female | 58% | 59% |
| Race/ethnicity |  |  |
| White non-Hispanics | 78% | 70% |
| Black non-Hispanics | 7% | 9% |
| Hispanics | 9% | 13% |
| Other | 5% | 8% |
| Any Difficulty with ADL | 32% | 35% |
| BMI (kg/m^2^) | 28.0 | 27.9 |
| Number of chronic conditions | 3.0 | 3.0 |
